# A methotrexate dashboard: integrating MTXPK.org into the electronic health record to facilitate model-informed care for pediatric patients receiving high-dose methotrexate

**DOI:** 10.1093/jamiaopen/ooag057

**Published:** 2026-04-16

**Authors:** Zachary L Taylor, Nieko Punt, Chris Jenkinson, Bill Vidonish, Jennifer Young, Maureen M O’Brien, Laura B Ramsey

**Affiliations:** Division of Translational and Clinical Pharmacology, Cincinnati Children’s Hospital Medical Center, Cincinnati, OH, 45229, United States; Center for Blood Diseases Institute, Cincinnati Children’s Hospital Medical Center, Cincinnati, OH, 45229, United States; Department of Pediatrics, University of Cincinnati, Cincinnati, OH, 45267, United States; Medimatics, Maastricht, 6211, The Netherlands; Department of Clinical Pharmacy and Pharmacology, University of Groningen, University Medical Center Groningen, Groningen, 9700 RB Groningen, The Netherlands; Department of Information Services, Cincinnati Children’s Hospital Medical Center, Cincinnati, OH, 45229, United States; Department of Information Services, Cincinnati Children’s Hospital Medical Center, Cincinnati, OH, 45229, United States; Division of Pharmacy, Cincinnati Children’s Hospital Medical Center, Cincinnati, OH, 45229, United States; Department of Pediatrics, University of Colorado Anschutz Medical Campus, Denver, CO, 80045, United States; Division of Clinical Pharmacology, Toxicology & Therapeutic Innovation, Children’s Mercy Kansas City, Kansas City, MO, 64108, United States; Department of Psychiatry and Behavioral Neuroscience, University of Cincinnati, Cincinnati, OH, 45267, United States

**Keywords:** Clinical decision support, pediatrics, oncology, methotrexate, precision medicine

## Abstract

**Purpose:**

The MTXPK.org webtool facilitates model-informed supportive care and glucarpidase use in patients receiving high-dose methotrexate, but its reliance on manual data entry limits workflow integration. We aimed to highlight how translational informatics can advance, automate, and enhance the usability of clinical decision support tools by embedding MTXPK.org within the electronic health record (EHR).

**Methods:**

A human factors study with 6 clinical providers guided iterative prototype development. MTXPK.org was rebuilt within the EHR using a 3-tier architecture with Fast Healthcare Interoperability Resources-based data retrieval, automated pharmacokinetic modeling, and interactive visualization.

**Results:**

Two rounds of prototyping with task-based evaluations and contextual inquiry showed progressive improvements in information recognition and navigation. The final design achieved excellent usability, with a System Usability Scale score of 90.4 compared to 57 for the original MTXPK.org tool. The dashboard is now live, automating data entry and generating individualized pharmacokinetic profiles with interactive visualization.

**Conclusions:**

The Methotrexate Monitoring Tool is an integrated methotrexate dashboard that automates data entry, improves usability, and facilitates model-informed supportive care and glucarpidase use.

## Background

High-dose methotrexate (HDMTX) is an antifolate administered intravenously at high doses to effectively treat pediatric and adult patients with common malignancies. Unfortunately, patients receiving HDMTX often experience significant toxicities of the kidneys, the liver, and gastrointestinal tract.^1–3^ To reduce these toxicities, supportive care (including fluid hyperhydration and urine alkalization before and during the HDMTX treatment) is used to facilitate renal elimination of MTX.^1^^,^^2^ After the HDMTX infusion, folate supplementation is used to restart the folic acid cycle inhibited by methotrexate (MTX) to reduce the risk of toxic side effects. However, there is still considerable interpatient variability in the clearance of HDMTX with severely delayed MTX clearance seen in 1-10% of HDMTX administrations.^2^

Glucarpidase is a US Food and Drug Administration (FDA)-approved exogenous enzyme that serves as a rescue agent for HDMTX overdose for patients with delayed MTX elimination. Glucarpidase rapidly hydrolyzes MTX into 2 inactive metabolites that are eliminated via renal and nonrenal pathways.^2^^,^^4^ This rapid hydrolysis reduces plasma MTX concentrations by >90% within 15 minutes of administration.^2^ While effective, glucarpidase is only indicated when MTX concentrations are >2 standard deviations above the mean excretion curve specific for the given dose to avoid underexposure to MTX and risk of relapse.^5^ The appropriate interpretation of this indication and administration of glucarpidase was a challenge, given the specific need to know the expected excretion curve and the standard deviation. As a result, a glucarpidase consensus guideline was published to clarify the interpretation of the glucarpidase indication by presenting an algorithm of actionable MTX concentrations at select time points following HDMTX infusion of several common dosing regimens.^2^ Although beneficial, the consensus guideline was not dynamic and was unable to provide recommendations when sample times didn’t overlap with specified time points. A newer European-focused guideline for glucarpidase has recently been published with similar recommendations.^6^

The MTXPK.org webtool is a freely available clinical decision support tool that was launched in December 2019 and acts as a dynamic visual guide to facilitate optimal glucarpidase use.^7^^,^^8^ The tool relies on a pharmacokinetic model as the population priors and estimates individual parameters after a user enters an individual patient’s data. The tool uses Bayesian estimation of real-time patient data to generate visual and numerical data that users can interpret in tandem with the consensus guideline and FDA drug label to facilitate model-informed supportive care and optimize glucarpidase use in pediatric and adult patients receiving HDMTX. To use the MTXPK.org webtool, clients must manually enter all relevant demographics, treatment data, and time-dependent lab data, which could result in entry errors. Recent advances allow users to upload these data directly into the webtool; however, data files must be correctly formatted to be read into MTXPK.org, thus the potential for entry errors is still a concern. Clinicians have requested integration with the electronic health record (EHR) to allow for automated entry of patient data and provide a more seamless transition into their workflow.

The overall purpose of this manuscript is to highlight how translational informatics can advance, automate, and enhance the usability of clinical decision support tools within the EHR environment. Therefore, the objective was to integrate the MTXPK.org webtool with the EHR at a pediatric research hospital. To accomplish this, we conducted a human-centered design process, including a design sprint and iterative prototype testing with clinicians, to guide development and usability of the EHR-integrated Methotrexate Monitoring Tool. Herein, we describe the conceptualization, design, system architecture, and pharmacokinetic model comprising the Methotrexate Monitoring Tool within the EHR.

## Methods

### Conceptualization and prototype development

A structured human-centered design process was conducted by POMIET LLC (Miamisburg, Ohio) in collaboration with Cincinnati Children’s to guide development of the Methotrexate Monitoring Tool. Six self-selected clinical providers (4 oncologists, 1 nurse practitioner, and 1 clinical pharmacy specialist) were recruited through an open call distributed to relevant oncology and pharmacy divisions at Cincinnati Children’s and volunteered their time to participate in a design sprint composed of discovery interviews, iterative prototype evaluations, and usability testing. First, one-on-one contextual inquiry interviews were conducted to understand clinician workflows related to high-dose methotrexate and supporting tools. Table 1 summarizes the aggregated themes derived from pooled user feedback across these sessions. Insights from these interviews informed the iterative creation of 2 no-code wireframe prototypes, which were evaluated by clinicians through standardized use case scenarios and qualitative feedback collected via contextual inquiry. Three scenarios were applied to each prototype: (1) sensemaking and decision-making with the dashboard, (2) recognition of methotrexate concentrations deviating from expected norms, and (3) decision-making related to medication administration. Each scenario was evaluated using task completion ratings, scored on a 5-point Likert scale (1 = immediate completion; 5 = unable to complete), with scores <2.0 considered usable. Scores were grouped into ‘Information Recognition’ and ‘Navigation.’ Feedback from the first round of evaluations guided the development of an interactive prototype, which was tested in a second round of user sessions (Table 1). Finally, the MTXPK.org tool and the final Methotrexate Monitoring Tool were evaluated quantitatively using the System Usability Scale (SUS), a validated 10-item instrument scored from 0 to 100, and qualitatively using contextual inquiry.^9^

**Table 1:** Prototype development and user feedback.

MTXPK.org	Prototypes	Methotrexate monitoring tool
**Data entry**		
Cumbersome, and time-consuming to enter patient data. Could be prone to errors.	Appreciated automated data entry. Recognized immediate benefit of an automated process.	Seamless integrated automated data entry allowed for timely use of the tool. Accelerated navigation.
**Multiple administrations**		
Unable to view prior methotrexate administrations. Prevented direct comparison of administrations.	Iterative designs settled on a drop- down menu for quick toggle between administrations. A graphical overlay had immediate benefit to healthcare providers.	Ability to overlay multiple concentration-time curves or toggle to specific administration dates accelerated information recognition and navigation.
**Reporting information**		
Documentation is great, but limited to the data entry fields. No direct reporting of baseline values or treatment details.	Additional documentation (eg, treatment details, baseline lab values) have benefit if easy to locate.	Patient data is quickly captured by the tool. Additional documentation is readily available.
**Leucovorin**		
Does not include leucovorin nomograms.	Reporting an institution’s leucovorin nomogram has immediate benefit to healthcare providers	Leucovorin nomograms are displayed by the Presentation Tier and can be toggled on/off as needed. Displaying this information accelerated information recognition.
**Integration with the electronic health record (EHR)**		
Tool is not available in EHR environment.	Access to the MTXPK.org web tool within the normal clinical workflow has immediate benefit.	Seamless integration enables access to the tool within the EHR environment.

### Dashboard design and system architecture

**Figure 1:**
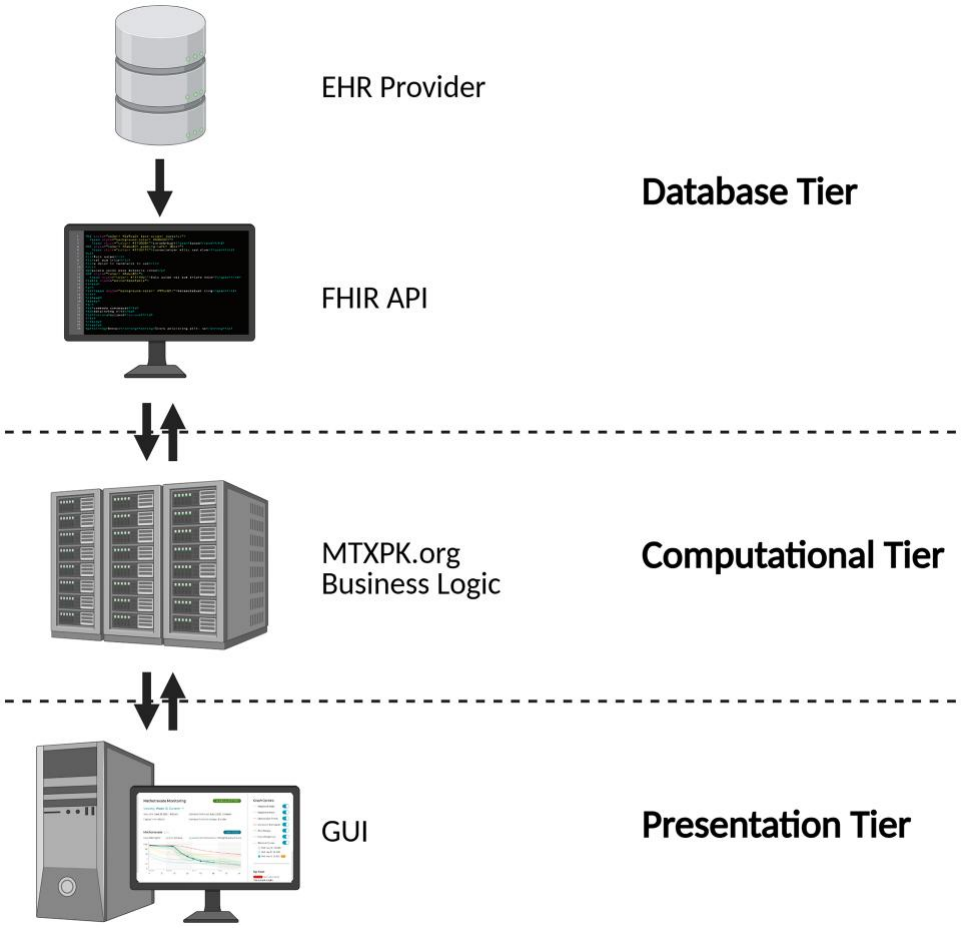
Schematic of methotrexate monitoring tool integration with the electronic health record. Abbreviations: EHR, electronic health record; FHIR, Fast Healthcare Interoperability Resources; GUI, graphical user interface. Figure created using BioRender.

The final prototype from the design sprint guided the development of the Methotrexate Monitoring Tool within the EHR (Figure 1). The tool was implemented using a 3-tier architecture.

#### The database tier

The Database Tier is the backend of the tool that comprises the EHR provider and houses the data recorded by clinical providers during clinical care. Nothing new was added to the clinical workflow to populate the tool. Upon accessing a patient’s chart in the EHR (Epic Hyperspace, Epic Systems Corporation), healthcare providers can navigate to a drop-down menu that includes a link to the “Methotrexate Monitoring Tool.” This link serves as a gateway to the tool, which opens an external window, providing an uninterrupted workflow within the EHR environment. The tool is implemented as a web-based application, with a React-based user interface and .NET backend services, enabling real-time dashboard rendering within the clinical workflow. The tool is then engineered to interact seamlessly with the EHR via Fast Healthcare Interoperability Resources (FHIR) application programming interface (API), which takes a snapshot of the current EHR environment then retrieves essential modeling information such as methotrexate dosing (eg, dose amount, infusion duration), patient demographics (eg, body surface area), and time-dependent laboratory results (eg, serum creatinine, albumin) for the prior year.

#### Computational tier

Patient-specific data retrieved from the Database Tier is fed to the MTXPK.org Business Logic, which accesses the Pharmacokinetic-Pharmacodynamic (PKPD) Modeling Engine and the PKPD Model Repository. Then, patient-level data is analyzed by the methotrexate pharmacokinetic model to generate patient-specific pharmacokinetic parameters (eg, clearance, CL [L/h/1.73m^2^], volume of distribution, V [L/1.73m^2^]) and concentration-time profiles.

#### Presentation tier

Outputs from the Computational Tier are communicated to the Presentation Tier, which generates an interactive concentration-time curve for the selected patient based on the modeled data retrieved from the EHR and presented by the graphical user interface (GUI). This colorblind-friendly curve graphically represents the patient’s individualized pharmacokinetics and provides interactive visual and numerical information that guides decision-making.

### Population pharmacokinetic model and evaluation

The MTXPK.org tool and Methotrexate Monitoring Tool call the PKPD Modeling Engine and the PKPD Model Repository, which houses a well-established 3-compartment population pharmacokinetic model of methotrexate with body surface area, serum creatinine, presence of hypoalbuminemia, presence of Down syndrome, and infusion duration as clinical variables associated with MTX clearance; and pleural effusion on volume of distribution.^8^ Model development and validation have been previously described; in brief, the model used US pediatric patients with a large range of methotrexate doses, infusion durations, diagnoses, body sizes, and renal function to provide the population priors for the Bayesian estimation of individual parameters.^8^ Model evaluations were performed to verify the tool’s data retrieval. The demographics (eg, body surface area), treatment characteristics (eg, methotrexate dose, infusion duration), clinical lab values (eg, methotrexate concentrations, serum creatinine levels), and fitted concentration-time profiles (including 95% confidence intervals and estimated time to elimination thresholds 0.2 µM and 0.1 µM) from the dashboard were compared to MTXPK.org for 10 patients with varied ages, dose amounts, and treatment protocols. Fitted concentration-time profiles were expected to be identical since both the dashboard and MTXPK.org call the same validated pharmacokinetic model in the MTXPK Business Logic (Figure 1).

## Results

### Prototype development

Findings from the design sprint and prototype evaluations informed the development and integration of the MTXPK.org tool within the EHR environment. The initial no-code wireframe prototype was developed from contextual inquiry interviews and emphasized consolidation of methotrexate data into a single dashboard. It received positive feedback and favorable task completion ratings for information recognition (mean = 1.29, median = 1); however, navigation was less efficient (mean = 1.9, median = 1.8) due to difficulty locating prior methotrexate administrations. The second iterative prototype incorporated a more interactive GUI and streamlined access to patient-specific information, which was reflected in qualitative feedback (Table 1) and improved task completion ratings for both information recognition (mean = 1.20, median = 1) and navigation (mean = 1.0, median = 1.0). These standardized use case scenarios and qualitative feedback collected via contextual inquiry guided the development of the Presentation Tier of the dashboard.

### Dashboard evaluation

MTXPK.org did not perform well during the quantitative evaluation, with a SUS score of 57, below the average benchmark of 68. The primary limitation was the lack of automated data entry, which increased task completion time; however, participants still expressed overall support for the MTXPK.org tool. In contrast, the final Methotrexate Monitoring Tool prototype achieved a SUS score of 90.4, reflecting excellent usability. Automation of data entry and seamless EHR integration, executed by the Database Tier, enabled clinicians to complete tasks efficiently, with additional qualitative feedback highlighting improvements in workflow integration (Table 1). Verification of implementation equivalence confirmed that the EHR-integrated dashboard accurately retrieved demographic, dosing, and laboratory data from the EHR. Execution of the Computational Tier reproduced pharmacokinetic outputs identical to those generated by MTXPK.org. No discrepancies in data handling, graphical display, or modeled concentration–time profiles were observed.

### Dashboard architecture

**Figure 2:**
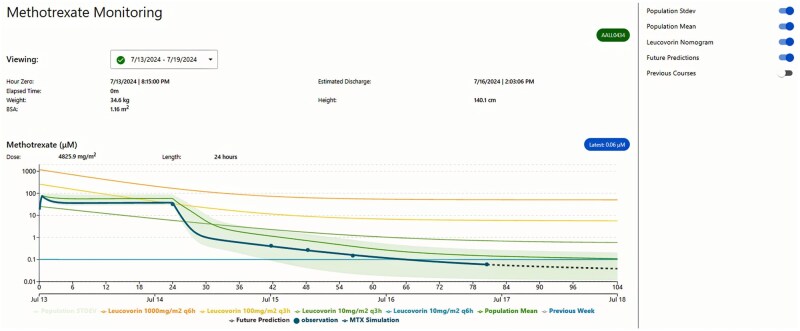
Representative image of the methotrexate monitoring tool launched inside the electronic health record. Patient A received a 5 g/m^2^ methotrexate dose over 24 hours (10% given as a bolus over the first 30 minutes, then 90% given as a continuous infusion over the following 23.5 hours). The Presentation Tier provides the user with a customizable and integrated system that supports physician decision-making and ongoing therapeutic drug management practices.

The Methotrexate Monitoring Tool is live within the EHR at Cincinnati Children’s (Figure 2), demonstrating the successful application of translational informatics principles to automate and enhance the usability of clinical decision support tools through multidisciplinary collaboration and user-centered design. The Presentation Tier displays a GUI designed using feedback from our self-selected clinical providers, and includes access to the patient’s methotrexate concentrations, Bayesian estimated concentration-time curve, forecasted Bayesian estimated concentration-time curve, the population’s concentration-time curve, 2 standard deviations above and below the population curve, and the institutional leucovorin nomogram. The interactive display allows users to toggle graph options that personalize the display settings and provide time-specific pharmacokinetic data by hovering over the graph.

## Discussion

The MTXPK.org clinical decision support tool facilitates model-informed supportive care and glucarpidase use for pediatric and adolescent/young adults receiving high-dose methotrexate for the treatment of malignant conditions. Users have noted that data entry for MTXPK.org can be cumbersome and susceptible to errors, and its lack of integration limits its use in conventional workflow.

Herein, we describe the development of the Methotrexate Monitoring Tool, which is an EHR-integrated clinical decision support dashboard designed to automate the data entry process for the embedded MTXPK.org modeling platform. This work exemplifies how translational informatics can advance, automate, and enhance the usability of clinical decision support tools within the EHR environment through multidisciplinary collaboration and the integration of user feedback.

The dashboard’s architecture allows for seamless integration across the system (Figure 1), similar to prior dashboards and commercially available software.^10–14^ The FHIR code successfully retrieves the required demographics, treatment data, and time-dependent labs from the EHR for automated input into the MTXPK.org Business Logic, which houses the pharmacokinetic model, performs the pharmacokinetic analysis, and generates the outputs to the GUI.^7^ The GUI provides an interactive, EHR-integrated version of MTXPK.org with dynamic visual and numerical feedback and population-based data overlaid with the institutional leucovorin nomogram to facilitate model-informed decision-making of supportive care interventions (Figure 2).

The automation of the data entry process into pharmacokinetic modeling fulfilled the clinical need to integrate this pipeline with the EHR (Table 1). We were able to address the providers’ needs throughout the iterative prototype development, ending with a final design that accelerated information recognition and navigation. The final design allowed for innovative additions to the MTXPK.org tool, including seamless comparison of prior methotrexate administrations and institutional leucovorin dosing nomograms, while maintaining core functions like estimated methotrexate exposure and estimated time to discharge. The clinical provider’s experience using the Methotrexate Monitoring Tool is a significant advancement compared to the MTXPK.org webtool. Clinical providers indicated their appreciation for the automation of data entry, which they felt would avoid data entry errors and minimize the time to achieve the desired results. They further noted that EHR integration enabled incorporation into routine decision-making, while the graphical features of MTXPK.org and GUI personalization were maintained (Table 1). Clinical providers also lauded the ability to readily overlay multiple methotrexate administrations for a single patient, and quickly toggle the display to specific administration dates. Since launch, the methotrexate monitoring tool has had robust engagement from more than 15 multidisciplinary users at Cincinnati Children’s, including physicians, advanced practice providers, and clinical pharmacists, accessing the tool 40-50 times per month. The majority of oncology providers report using the tool with every HDMTX course and value most the ability to estimate time of clearance and compare previous courses. Additionally, providers find the tool to be a useful visual aid in guiding patient and family expectations for clearance.

While the Methotrexate Monitoring Tool has been successfully launched at a pediatric research hospital, a major limitation is the potential for interoperability across institutions, even with the same choice in EHR provider.^10^^,^^11^ FHIR code used to retrieve the data required for pharmacokinetic modeling (eg, medication identifiers for methotrexate, infusion duration, body surface area) was specific to work within the Epic EHR at Cincinnati Children’s and has limited cross-institutional integration. Working directly with EHR providers could overcome this limitation. Our FHIR code is also sensitive to available data fields; changes to medication identifiers or clinical lab values (ie, serum creatinine vs plasma creatinine) could result in missing data that prevents the pharmacokinetic model from running. Additionally, the same 6 self-selected participants were engaged across all design phases; this intentional continuity, while limiting generalizability, strengthened iterative feedback from experienced MTXPK.org users and end-user champions of clinical decision support. Lastly, formal usability evaluation relied primarily on the SUS, and qualitative feedback was provided in aggregated summaries rather than verbatim transcripts or recordings. While these approaches enabled rapid-cycle prototype refinement, future studies incorporating broader user sampling, objective task-completion metrics (eg, time-to-decision analyses), longitudinal usage analytics, and cross-institutional evaluation would further enhance generalizability, strengthen evidence for operational adoption, and support sustained clinical uptake.

## Conclusion

The Methotrexate Monitoring Tool is an EHR-integrated extension of MTXPK.org designed to improve usability by automating patient data entry, reducing the chance for entry errors, and minimizing the time required to use the tool. This tool showcases the power of collaboration across researchers, clinical informaticists, and clinical providers to create translational tools that improve patient outcomes and adds to the growing number of available EHR-integrated dosing dashboards used in routine clinical care at CCHMC.^12–14^

## Data Availability

The data underlying this article will be shared on reasonable request to the corresponding author.

## Previous presentations

American Conference of Pharmacometrics, November 10-13, 2024.
